# Engineered three-dimensional rabbit oral epithelial–mesenchymal–muscular hybrid sheets

**DOI:** 10.1038/ijos.2016.16

**Published:** 2016-06-24

**Authors:** Shigeki Yamane, Kazunari Higa, Takashi Umezawa, Masamitsu Serikawa, Jun Shimazaki, Shinichi Abe

**Affiliations:** 1 Department of Anatomy, Tokyo Dental College, Tokyo, Japan; 2 Department of Ophthalmology/Cornea Center, Tokyo Dental College, Chiba, Japan

**Keywords:** mesenchymal stem cell, multi-differentiation, myoblast, oral mucosa, three-dimensional culture

## Abstract

Regenerative muscles are required for swallowing and mastication, and are important for functional recovery from diseases involving oral muscular defects. Therefore, we generated three-layer hybrid sheets, similar to oral mucosal structures containing submucosal muscles, using rabbit oral mucosa epithelial, mesenchymal, and myoblastic progenitor cells, and examined the structural proteins. Each cell type was obtained from rabbit oral mucosa using enzymatic digestion. Isolated mesenchymal and myoblastic cells were multi-differentiated into osteoblasts, adipocytes, and chondrocytes or myotubes. Isolated epithelial cells were cultured on collagen gels containing isolated mesenchymal cells for 2 weeks, and these epithelial–mesenchymal cell sheets were laminated onto myoblastic cell sheets. The engineered hybrid sheets were multi-stratified in the epithelial and myoblastic layers in a time-dependent manner, expressing intermediate cytoskeletal filament proteins of epithelium and muscle. Hybrid sheets also expressed extracellular matrix basement membrane proteins. Immature cell markers for epithelial and myoblastic cells were observed continuously in hybrid sheet cultures. We established engineered three-dimensional rabbit oral mucosa hybrid sheets containing each immature cell type *in vitro*.

## Introduction

Mucous membranes line the oral cavity, pharynx, and esophagus, which are involved in mastication and swallowing. Directly beneath the mucosal epithelium is a layer of muscles that includes the buccinator muscles and the pharyngeal constrictors, among others. This continuous muscle layer has a crucial role in oral cavity and pharynx function. Antebrachial flaps and rectus abdominis flaps have often been used for reconstruction following the removal of malignant tumors from the oral cavity, such as tongue cancers or pharyngeal cancer, and have enabled recovery of the minimally required shape and function of the oral cavity.^1, 2, 3^ However, recovery of muscle function required for swallowing and chewing is difficult with conventional methods employing grafted tissue. Moreover, postoperative scarring represents a significant burden for patients.

Cell sheet engineering has progressed in recent years, and reconstruction techniques following the removal of malignant tumors, such as esophageal or stomach cancers, using oral mucosal epithelial cell sheets created with the patient's own cells are gradually being applied in clinical practice.^4, 5, 6, 7, 8, 9^ However, no cases of reconstruction have been presented that includes subepithelial connective tissue and muscles. In addition, reconstruction using oral mucosal epithelial cell sheets has not been performed following tongue or pharyngeal cancer. Given that the tongue and pharynx have a central role in swallowing and chewing, grafts working in concert with tissue *in vivo* are essential for increasing patients' quality of life after surgery. We therefore set out to develop cultured grafts using three-layered sheets created from a hybrid of epithelial, mesenchymal, and muscular cell sheets. Among studies of tissue regeneration via cell sheet engineering, there have been no examples of the creation of layered sheets of tissue cells extending past the germ layer that have embryologically different origins.

We previously established a culture model of rabbit oral mucosal epithelial sheets,^10^ and therefore isolated and cultured epithelial cells, mesenchymal cells, and myoblasts from the oral mucosal tissue of Japanese domestic rabbits and created cell sheets. We reproduced *in vitro* the three-layered structure that is observed *in vivo*, and compared and examined the expression of cytoskeletal and adhesive proteins that are essential for maintaining this structure.

## Materials and methods

### Antibodies

Mouse monoclonal antibodies for keratin (K) 4, K13, laminin, and desmin were purchased from DBS (6B10; Pleasanton, CA, USA), Progen (2D7; Heidelberg, Germany), Cosmo Bio (NU-01-LA3; Tokyo, Japan), and Santa Cruz Biotechnology (RD301; Santa Cruz, CA, USA). Anti-collagen type IV goat polyclonal antibody was purchased from SouthernBiotech (Birmingham, AL, USA). Fluorescein isothiocyanate (FITC), rhodamine-, and Cy3-conjugated secondary antibodies were purchased form Jackson ImmunoResearch Laboratories (West Grove, PA, USA) or Chemicon International (Temecula, CA, USA).

### Preparation of rabbit oral tissues

Female Japanese white rabbits (2.5 kg each) were purchased from Japan CLEA (Tokyo, Japan). Rabbit oral mucosal tissues were prepared from oral cavities after anesthetizing them with 100 mg·kg^−1^ pentobarbital sodium (Kyoritsu Seiyaku, Tokyo, Japan) and killing using 1 mol·L^−1^ potassium chloride (Wako, Osaka, Japan). All experimental procedures and protocols were approved by the Animal Care and Use Committee of Tokyo Dental College (approval number: 250105) and conformed to the National Institutes of Health Guide for the Care and Use of Laboratory Animals.

### Isolation of oral mucosal epithelial cells

Rabbit mucosal specimens were dissected and submucosal connective tissues, such as adipose and muscle tissues, were removed with scissors. The epithelium was cut into small pieces and washed several times in a 1:1 (*V*/*V*) mixture of Dulbecco's modified Eagle's medium (DMEM; Invitrogen, Grand Island, NY, USA) and Ham's F12 (Invitrogen, Grand Island, NY, USA) containing 5 g·mL^−1^ gentamicin (Invitrogen, Grand Island, NY, USA), and 0.25 g·mL^−1^ amphotericin-B (Sigma-Aldrich, St. Louis, MO, USA). Epithelial sheets were isolated using 1.2 U·mL^−1^ dispase II (Roche, Mannheim, Germany) at 4 °C overnight, as described previously.^11^ Dispersed epithelial sheets were treated with trypsin–ethylene diaminetetraacetic acid (EDTA) for 10 min to produce cell suspensions. The oral mucosal epithelial cell suspensions were used for organotypic co-cultures with isolated oral mucosa mesenchymal cells in collagen gel, and functioned as alternating feeder cells during epithelial sheet development.

### Isolation of oral mucosal mesenchymal cells

Oral mucosal connective tissues from the remnants of the dispased epithelial sheets were used to isolate oral mucosal mesenchymal cells. Oral mucosal connective tissues were treated with 2 mg·mL^−1^ collagenase at 37 °C overnight and cultured in mesenchymal stem cell growth medium (Lonza Walkersville, Walkersville, MD, USA). To isolate rabbit oral mucosal mesenchymal cells, we cultured single cells (8.0 × 10^3^ cells per ml to minimize cell aggregation^12^) of amplified rabbit oral mucosa mesenchymal cells with 0.8% methylcellulose in advanced DMEM containing 10% fetal calf serum (FCS) on low-adhesive plates (HydroCell; CellSeed, Tokyo, Japan) to avoid cell attachment. After 2 weeks at 37 °C in humidified air with 5% CO_2_, clusters were formed from single cells. Clusters were replated on adhesive plates and amplified by explant adhesive culture. To examine the characteristics of amplified mesenchymal cells, we attempted to induce differentiation into osteoblasts, adipocytes, and chondrocytes of mesenchymal lineages using differentiation media (osteogenic, adipogenic, and chondrogenic induction medium; Lonza Walkersville, Walkersville, MD, USA) for 2 weeks according to the manufacturer's instructions.

### Isolation of oral mucosal myoblasts

Muscle tissue was separated from rabbit mucosal specimens, cut into small pieces with scissors, and treated with 2.5% trypsin for 2 h at 37 °C. Isolated cells from dissociated tissue were cultured in advanced DMEM with 10% FCS for 30 min at 37 °C, and non-adhesive cells were transferred to a fresh 1% gelatin-coated flask (Becton and Dickinson, Sparks, MD, USA) using differential adhesion rates to remove non-myoblastic cells, such as fibroblasts.^13, 14, 15^ These cells could be subcultured over 20 times using differential adhesion rates on 1% gelatin-coated flasks.

### *In vitro* differentiation

When cells became semi-confluent, they were washed in phosphate-buffered saline (PBS; pH 7.2) and incubated with TrypLE (Invitrogen, Grand Island, NY, USA) for 5 min at 37 °C. The collected cells were seeded at a density of 5.0 × 10^3^ cells per cm^2^ in four-well chamber slides (LAB-TEK, Nalge Nunc, Rochester, NY, USA). For chondrogenesis using pellet cultures, 2.5 × 10^5^ cells were placed in 15-ml polypropylene tubes (BD Falcon, Franklin Lakes, NJ, USA) and collected by centrifugation at 440*g* for 5 min at 4 °C.

Isolated cells were cultured in advanced DMEM with 10% FCS until they reached semi-confluency to induce mesenchymal cells. For osteogenic induction, the cultures were further grown in osteogenic induction medium (Lonza Walkersville, Walkersville, MD, USA) containing dexamethasone, ascorbate mesenchymal cell growth supplement (MCGS), *L*-glutamine, β-glycerophosphate, and gentamicin/amphotericin-B (GA)-1000 (Lonza Walkersville, Walkersville, MD, USA) for 3 weeks. For adipogenic induction, the cultures were further grown in adipogenic induction medium (Lonza Walkersville, Walkersville, MD, USA) containing human recombinant insulin, *L*-glutamine, MCGS, dexamethasone, indomethacin, 3-isobutyl-methyl-xanthine, and GA-1000. Control groups were grown in adipogenic maintenance medium (Lonza Walkersville, Walkersville, MD, USA) containing human recombinant insulin, *L*-glutamine, MCGS, and GA-1000 for 3 weeks. For chondrogenic induction, the cultures were further grown in complete chondrogenic induction medium (Lonza Walkersville, Walkersville, MD, USA) containing dexamethasone, ascorbate, insulin–transferrin–selenium (Lonza Walkersville, Walkersville, MD, USA) supplement, GA-1000, sodium pyruvate, proline, *L*-glutamine, and transforming growth factor (TGF)-β3 (Lonza Walkersville, Walkersville, MD, USA). Control groups were grown in incomplete chondrogenic induction medium without TGF-β3. The medium was changed three times a week and cultures were analysed after 3 weeks.

### Alizarin red S staining

Cultured cells were fixed in 70% cold ethanol for 10 min and rinsed with distilled water, after which they were stained with Alizarin red S solution (Roche, Mannheim, Germany) for 10 min at room temperature (RT). Finally, the cells were rinsed with deionised water and observed using a microscope.

### Oil red O staining

Cells cultured in chambers were fixed in 4% cold paraformaldehyde for 10 min and rinsed with 60% isopropyl alcohol (Wako, Osaka, Japan). An amount of 200 mg of Oil Red O (Sigma-Aldrich, St. Louis, MO, USA) was dissolved in 10 mL 60% isopropyl alcohol and filtered. Fixed cells were stained with a 2% Oil red O solution for 5 min at RT, after which they were rinsed with deionized water, counterstained with hematoxylin (Wako, Osaka, Japan) for 15 min, and observed using a microscope.

### Safranin O stain

The induced micromasses were frozen in Tissue-Tek optimum cutting temperature (OCT) compound (Sakura Finetek, Tokyo, Japan), sliced into 5-μm-thick sections, and fixed in 10% formalin solution (Wako, Osaka, Japan) for 10 min. Each section was then rinsed with deionized water and stained with 6% Safranin O solution (Chroma, Münster, Germany) for 5 min at RT. The sections were then rinsed with deionized water, counterstained with hematoxylin for 2 min, and observed using a microscope.

### Organotypic co-cultures for cell sheet production

**Figure 1:**
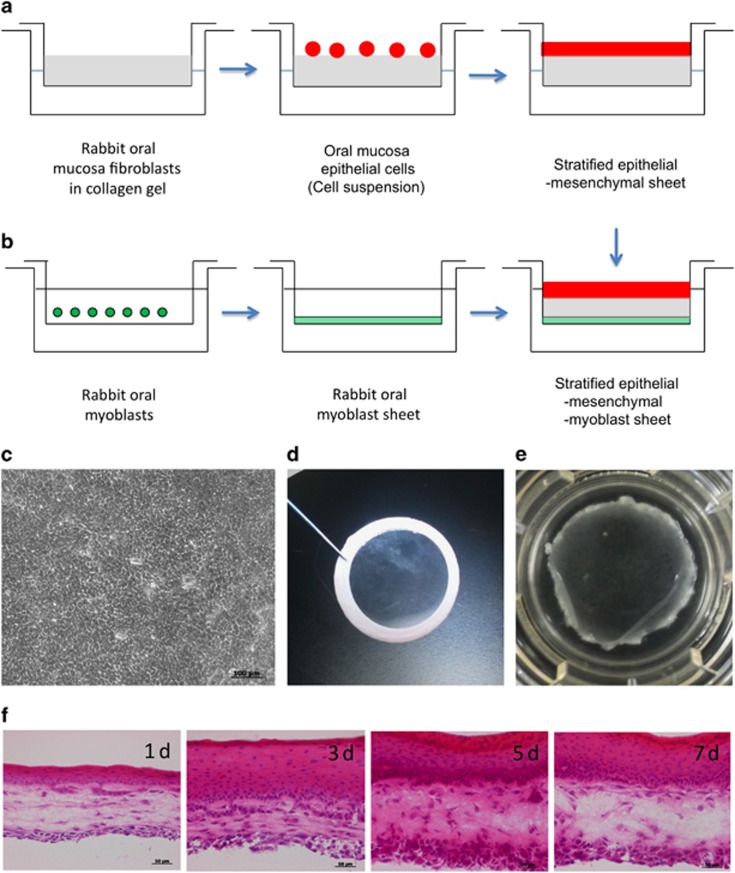
**Cultivation method for rabbit oral hybrid sheets**. (**a**) Rabbit oral mucosa epithelial cells were collected and seeded on collagen-coated inserts containing rOMMCs. After 1–2 weeks of cultivation, cells were allowed to stratify at the air–liquid interface for 4 days. (**b**) rOMYCs were seeded on inserts until confluent and used to generate laminated epithelial–rOMMCs sheets. (**c**) A phase-contrast image of stratified epithelial–rOMMCs sheets. Scale bars, 100 μm. (**d**) Stratified epithelial–rOMMCs sheets carried by ring membrane support onto rOMYCs sheets. (**e**) Photograph of a laminated epithelial–rOMMCs sheet on a rOMYCs sheet. (**f**) Hematoxylin and eosin staining of rabbit hybrid sheets after 1, 3, 5, and 7 days. Scale bars, 50 μm. rOMMCs, rabbit oral mucosa mesenchymal tissues; rOMYCs, rabbit oral myoblasts.

For the preparation of an artificial oral mucosa, a suspension of cultured mesenchymal cells in advanced DMEM supplemented with 10% FCS was added to the collagen solution (Cellmatrix, Nitta Gelatin, Osaka, Japan). We optimized the culture conditions, specifically mesenchymal cell density in the collagen gel and mixture volume in the culture insert (Transwell, Costar Corning, Corning, NY, USA). The final concentration of mesenchymal cells was 6.25 × 10^4^ cells per ml 0.21% type IA collagen gel and 800 μL per well of the mixture was added onto a culture insert. The mixture was allowed to gel at 37 °C for 30 min in an atmosphere of 5% CO_2_. The isolated oral mucosal epithelial cells were gently overlaid onto the surface of the equilibrated gel and cocultured with supplemented hormonal epithelial medium (SHEM) containing DMEM/F12 (Invitrogen, Grand Island, NY, USA), 10 ng·mL^−1^ human epidermal growth factor (Invitrogen, Grand Island, NY, USA), 5 μg·mL^−1^ insulin (Sigma-Aldrich, St. Louis, MO, USA), 100 ng·mL^−1^ to 0.25 μg·mL^−1^ isoproterenol (Sigma-Aldrich, St. Louis, MO, USA), 1.3 mg·mL^−1^ triiothyronine (Sigma-Aldrich, St. Louis, MO, USA), 10% fetal bovine serum, 100 U·mL^−1^ penicillin (Wako, Osaka, Japan), 100 μg·mL^−1^ streptomycin (pH 7.2; Wako, Osaka, Japan), and 666 kU·mL^−1^ aprotinin (Wako, Osaka, Japan), which inactivates nonspecific proteases that degrade collagen, for 2 weeks at 37 °C in an atmosphere of 5% CO_2_. The gel surface was raised to the air–liquid interface to induce stratification of keratinocytes by lowering the medium level for 4 days (Figure 1a). Myoblast-like cells were prepared to coordinate with oral mucosa epithelial and mesenchymal co-constructs. Myoblast-like cells were seeded at a density of 1.1 × 10^4^ cells per cm^2^ on a culture insert (Transwell, Costar Corning, Corning, NY, USA) and cultured in advanced DMEM with 10% FCS for 2–4 days (Figure 1b). The co-constructs were laminated onto prepared myoblast-like cells using donut-shaped filter paper (Figure 1d). Samples were collected at 1, 3, 5, and 7 days for immunohistochemistry, reverse transcriptase polymerase chain reaction (RT-PCR), and western blotting to assess changes over time in the three-layer cultures.

### Immunohistochemistry

The three-layer specimens were mounted with OCT, and 5- or 16-μm frozen sections were cut for hematoxylin and eosin staining and immunostaining. Frozen sections were fixed for 10 min in 2% paraformaldehyde (Wako, Osaka, Japan) before blocking. Sections were blocked by incubation with 10% normal donkey serum (Chemicon International, Temecula, CA, USA) and 1% bovine serum albumin (Sigma-Aldrich, St. Louis, MO, USA) for 1 h at RT. Primary antibodies to desmin (1:300), K4 (1:20), K13 (1:10), collagen type IV (1:400), and laminin 3 (1:50) were applied and incubated for 90 min at RT, followed by incubation with FITC-, rhodamine-, or Cy3-conjugated secondary antibodies. After three washes with PBS, the sections were incubated with 1 mg·mL^−1^ 4,6-diamidino-2-phenylindole (Dojindo Laboratories, Tokyo, Japan) at RT for 5 min. Finally, the sections were washed twice in PBS and coverslipped using an aqueous mounting medium containing an anti-fading agent (Fluoromount/Plus; Diagnostic Biosystems, Pleasanton, CA, USA). Images were obtained using a florescence microscope (Axioplan2 imaging; Carl Zeiss, Thornwood, NY, USA) and a laser scanning confocal microscope (LSM510; Carl Zeiss, Thornwood, NY, USA).

### RT-PCR analysis

Total RNA was isolated from mesenchymal and myoblast-like cells using the SV Total RNA Isolation System (Promega, Madison, WI, USA) according to the manufacturer's recommendations. Complementary DNA (cDNA) was prepared from each total 5 μg RNA sample by incubating a 25 μL mixture containing 0.25 mol·L^−1^ dithiothreitol, 5 × reaction buffer, RNase inhibitor, and avian myeloblastosis virus reverse transcriptase (Takara Bio, Shiga, Japan) for 1 h at 41 °C. This cDNA was used as a template for PCR amplification. Amplifications (0.5 μL cDNA in a total reaction volume of 50 μL) were performed at 95 °C for 1 s, at 52 °C for 30 s, and at 72 °C for 20 s (3 cycles); followed by 95 °C for 30 s, at 52 °C for 30 s, and at 72 °C 20 s (25 cycles) using a GeneAmp PCR System 9700 thermocycler (Applied Biosystems, Foster City, CA, USA). Primer sequences, reaction conditions, and the size of each product are listed in Table 1. The amplification of glyceraldehyde-3-phosphate dehydrogenase was performed in the same manner to evaluate the cDNA quality. Amplification products were separated by electrophoresis on 1.5% or 2.0% agarose gels.

### Western blot analysis

The three-layer specimens were dissociated with lysis buffer (contain 50 mmol·L^−1^ tris-(hydroxymethyl)-aminomethane (Tris)–HCl (pH 7.4), 150 mol·L^−1^ NaCl, 1% Nonidet P-40; Calbiochem, Darmstadt, Germany) and homogenized. Each sample was incubated for 40 min at 4 °C, and then centrifuged at 15 000 r·min^−1^ for 30 min at 4 °C. Protein concentrations in the supernatants were determined using the DC protein assay (Bio-Rad Laboratory, Hercules, CA, USA). All samples were then diluted in modified 2 × samples buffer (4 × NuPAGE LDS sample buffer; Invitrogen, Grand Island, NY, USA), 12% 2-mercaptoethanol (Wako, Osaka, Japan) in lysis buffer, and boiled. An amount of 30 mg of each sample was loaded on a 12% Bis-Tris gel (Novex NuPAGE; Invitrogen, Grand Island, NY, USA) and transferred onto polyvinylidene difluoride membranes (Millipore, Billerica, MA, USA). The membranes were blocked with diluted normal serum (Vectastain ABC Kit; Vector Laboratories, Burlingame, CA, USA) for 60 min at RT. The membranes were incubated with antibodies for desmin (1:100), K13 (1:25), collagen type IV (1:250), and β-actin (1:1 000, mAbcam8226; Abcam, Cambridge, UK) for 90 min at RT. After the membranes were washed three times in PBS, biotinylated secondary antibodies (Vector Laboratories, Burlingame, CA, USA) were added for 30 min at RT. Protein bands were visualized (Vectastain ABC Elite Kit; Vector Laboratories, Burlingame, CA, USA) with DAB (Vector Laboratories, Burlingame, CA, USA) as the substrate. The plot profiles of the bands were analysed with Image J software (National Institutes of Health, Bethesda, MD, USA). Statistical significance was evaluated using *t*-tests.

## Results

### Isolation of mesenchymal cells and myoblasts from oral mucosa and their analysis

**Figure 2:**
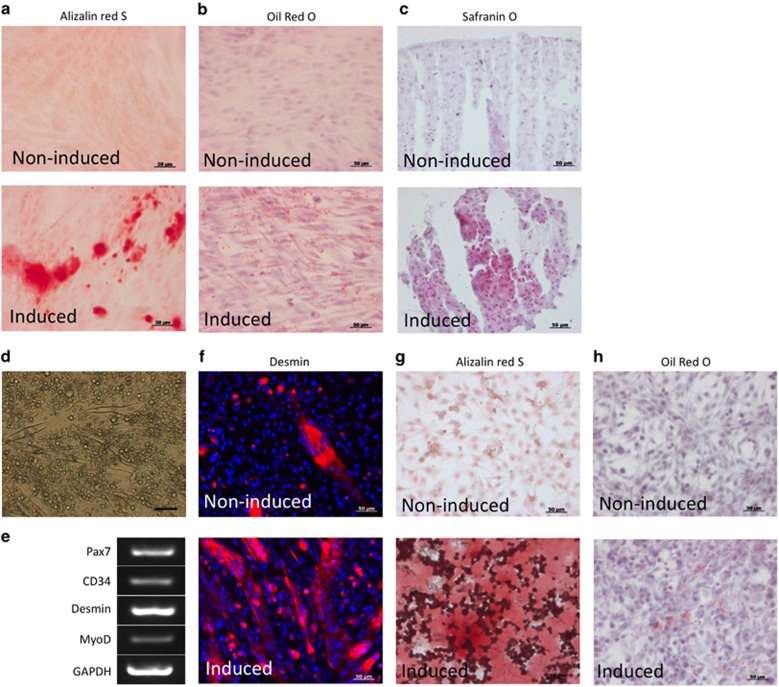
**Multi-differentiation of rOMMCs and rOMYCs**. (**a**) Alizarin red S staining of non-induced (upper panel) or osteogenic-induced (lower panel) rOMMCs. (**b**) Oil red O staining of non-induced (upper panel) or adipogenic-induced (lower panel) rOMMCs. (**c**) Safranin O staining of non-induced (upper panel) or chondrogenic-induced (lower panel) rOMMCs. Scale bars, 50 μm. (**d**) Phase-contrast image of high-density culture of rOMYCs. (**e**) Expression of Pax7, CD34, desmin, and MyoD by RT-PCR in high-density culture of rOMYCs. GAPDH was used as an internal control. (**f**) Immunocytochemistry of desmin (red) in high-density non-induced (upper panel) or myogenic-induced rOMYCs (lower panel). Nuclei were stained with 4,6-diamidino-2-phenylindole. (**g**) Alizarin red S staining in non-induced (upper panel) and osteogenic-induced (lower panel) rOMYCs. (**h**) Oil red O staining in non-induced (upper panel) and adipogenic-induced (lower panel) rOMYCs. Scale bars, 50 μm. GAPDH, glyceraldehyde-3-phosphate dehydrogenase; rOMMCs, rabbit oral mucosa mesenchymal tissues; rOMYCs, rabbit oral myoblasts.

Cells collected from rabbit oral mucosa mesenchymal tissues (rOMMCs) were successfully amplified from single cells by methylcellulose culture. Alizarin red-positive calcium deposition was observed in osteogenic-induced rOMMCs (Figure 2a). Oil red-positive lipid droplets were observed in adipogenic-induced rOMMCs, and cartilage mucin staining Safranin O-positive cells were observed in chondrogenic-induced rOMMCs (Figures 2b and 2c).

We were able to selectively culture rabbit oral myoblasts (rOMYCs) from cells isolated from submucosal muscle tissue by utilizing their low-adhesion property. High-density cultures of amplified rOMYCs, even after being subcultured 30 times, exhibited myotube-like structures and expressed MyoD and desmin, which are observed in myoblasts (Figures 2d–2f, upper). We also observed the expression of Pax7 and CD34, which are observed in comparatively undifferentiated rOMYCs (Figure 2e). Differentiation induced in a low-nutrient medium with 2% horse serum resulted in greater desmin expression (Figure 1f, lower). Alizarin red- and Oil red O-positive stained cells were observed in both osteogenic- and adipogenic-induced rOMYCs (Figures 2g and 2h).

### Three-layered oral mucosa fabrication *in vitro* and temporal tissue changes

We prepared a stratified epithelial sheet on collagen gel containing mesenchymal cells using an established method. By seeding isolated rOMMCs on collagen gels and culturing rabbit oral mucosa epithelial cell sheets on the surface, we were able to fabricate transparent two-layered sheets of multi-stratified epithelial cells and mesenchymal cells with a cobblestone appearance (Figures 1c and 1d). These two-layered sheets were then laminated onto myoblastic cell sheets to successfully create cohesive three-layered epithelial–mesenchymal–muscular cell sheets (Figure 1e). After lamination, multi-stratification progressed in the epithelial layer to ~20 layers after 7 days, and was also observed in the muscular layer from the fifth day onwards (Figure 1f).

### Temporal changes in epithelial structural proteins in three-layered sheets

**Figure 3:**
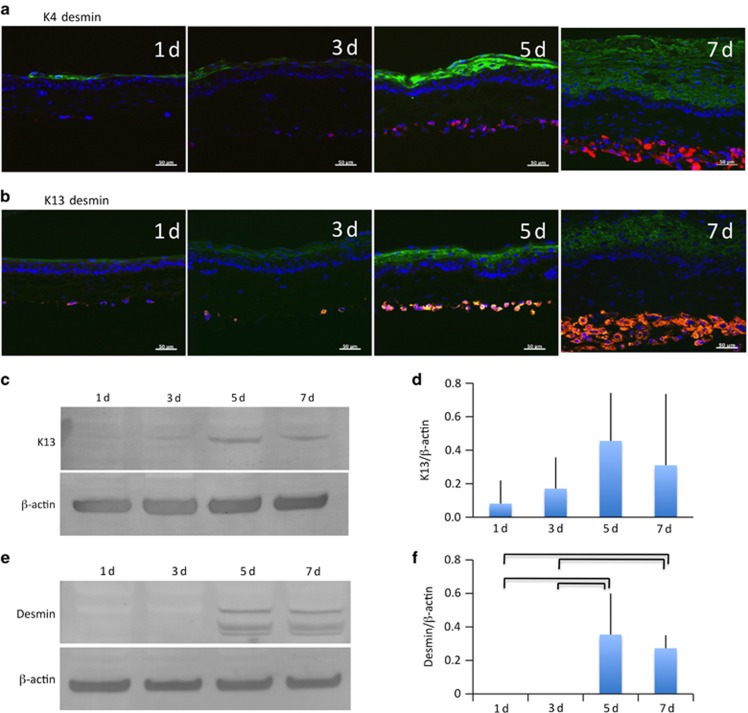
**Alteration of expressed epithelial and muscular structural proteins in hybrid sheets**. (**a**) Double staining of rabbit hybrid sheets after 1, 3, 5, and 7 days with K4 (green) and desmin (red). Nuclei were stained with DAPI. Scale bars, 50 μm. (**b**) Double staining of rabbit hybrid sheets after 1, 3, 5, and 7 days with K13 (green) and desmin (red). Nuclei were stained with DAPI. Scale bars, 50 μm. (**c**) K13 western blot of rabbit hybrid sheets after 1, 3, 5, and 7 days. β-Actin was used as an internal control. (**d**) Relative expression of K13 (K13/β-actin) protein (*n*=4). (**e**) Desmin western blot of rabbit hybrid sheets after 1, 3, 5, and 7 days. β-Actin was used as an internal control. (**f**) Relative expression of desmin (desmin/β-actin) protein (*P*<0.05, *n*=4). DAPI, 4,6-diamidino-2-phenylindole.

We used immunohistochemical staining to look for K4 and K13, which are unique cytoskeletal proteins present in mucosal epithelia, and observed both keratins distributed evenly throughout the epithelial tissue (Figure 3a and 3b). Western blotting was then performed to examine the changes in protein amounts.

Although the relative expression of K13 tended to increase over time, no significant difference was observed (Figure 3c and 3d). Desmin, which is a structural protein in muscle, was observed consistently over time and steadily increased in volume with increasing stratification (Figure 3a and 3b). Western blotting revealed that desmin protein levels significantly increased on days 5 and 7 *vs* day 1 (Figure 3e and 3f).

### Temporal changes in basement membrane adhesion proteins in three-layered sheets

**Figure 4:**
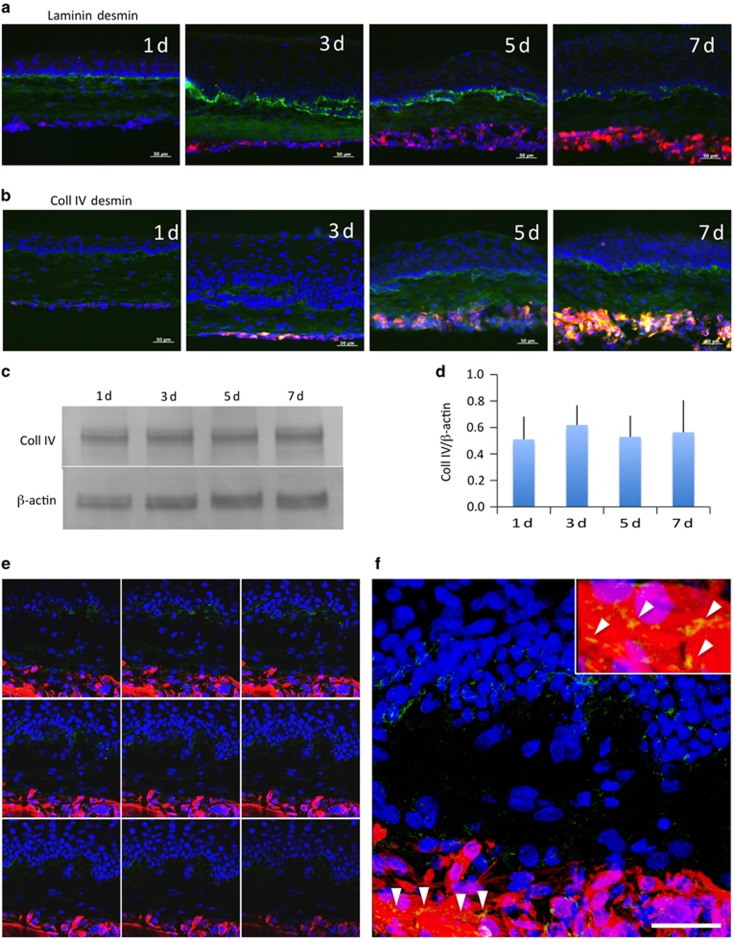
**Alteration of expressed basement membrane components in hybrid sheets**. (**a**) Double staining of rabbit hybrid sheets after 1, 3, 5, and 7 days with laminin (green) and desmin (red). Nuclei were stained with DAPI. Scale bars, 50 μm. (**b**) Double staining of rabbit hybrid sheets after 1, 3, 5, and 7 days with collagen type IV (Coll IV, green) and desmin (red). Nuclei were stained with DAPI. Scale bars, 50 μm. (**c**) Coll IV western blot of rabbit hybrid sheets after 1, 3, 5, and 7 days. β-Actin was used as an internal control. (**d**) Relative expression of Coll IV (Coll IV/β-actin) protein (*n*=4). (**e**) Laser scanning confocal images of a hybrid sheet after 7 days. All panels are 1 μm scanning images. Coll IV (green) was expressed in the epithelial basement membrane, the collagen gel layer containing rOMMCs, and in the desmin (red)-positive rOMYCs layer in all images. Nuclei were stained with DAPI. Scale bars, 20 μm. (**f**) Representative image of **e**. Coll IV was expressed in fused and multi-nucleated rOMYCs (arrowheads). Light upper insert is an enlarged image of the area depicted by arrowheads. DAPI, 4,6-diamidino-2-phenylindole; rOMYCs, rabbit oral myoblasts.

Immunohistochemical staining revealed the expression of collagen type IV (Coll IV), a basement membrane adhesive protein, in the epithelial basement membrane layer, collagen gel layer, and muscle layer, whereas the expression of laminin was observed only in the epithelial basement membrane layer (Figure 4a and 4b). Determination of temporal changes in Coll IV levels by western blotting did not reveal significant changes in Coll IV levels (Figure 4c and 4d).

### Observation of multi-stratified myoblasts by confocal laser microscopy

We performed three-dimensional analysis using confocal laser microscopy to observe multi-stratified myoblasts in the three-layer sheets. Complicated planar expression of Coll IV was observed between the epithelial–mesenchymal layer and mesenchymal–muscular layer (Figure 4e and 4f). Coll IV was expressed not only between the muscular layer and the connective tissue but also among myoblasts, and was observed around cell-fused myotube-like structures (Figure 4e, arrowheads).

### Analysis of gene expression in three-layered laminated sheets

**Figure 5:**
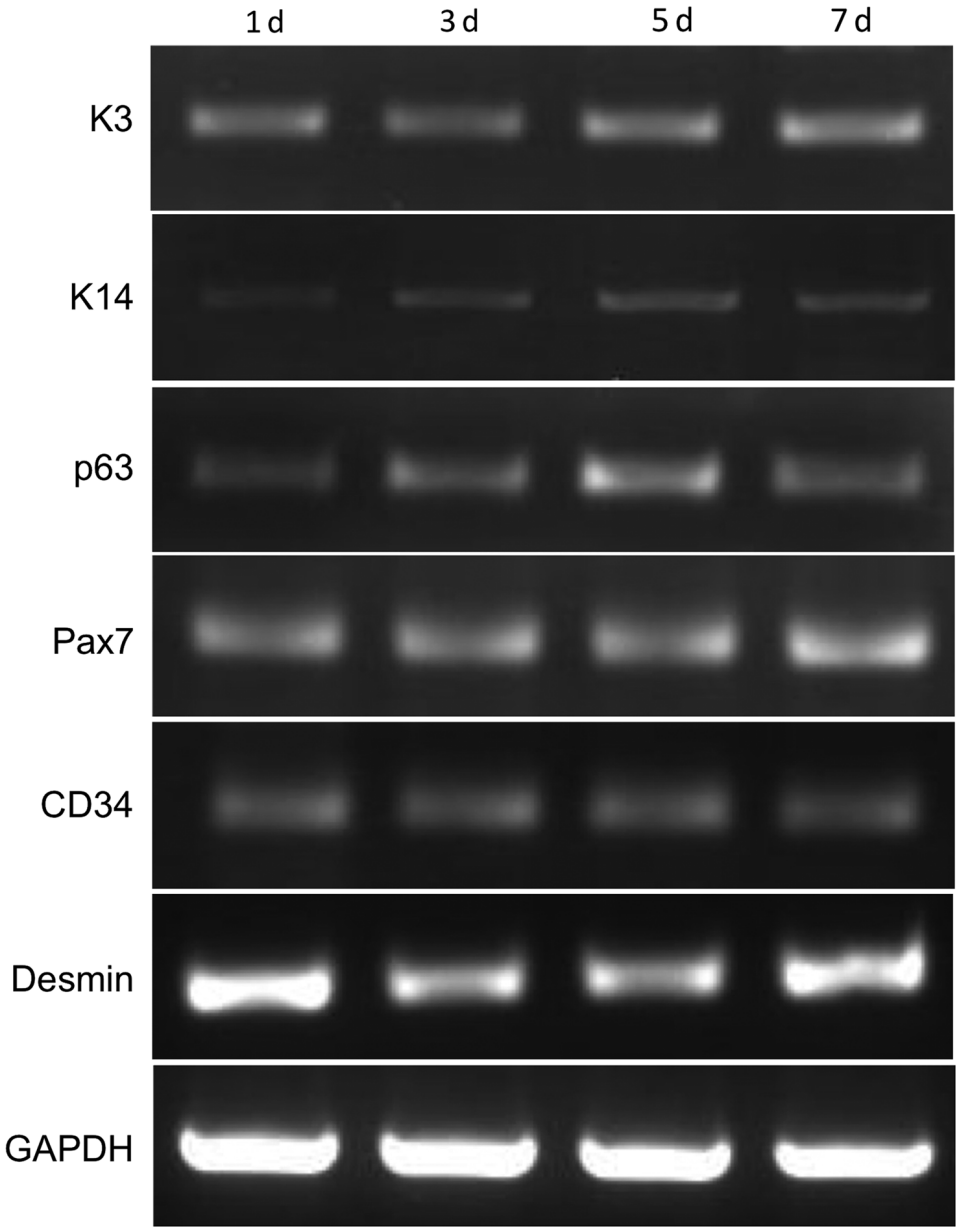
**Expression of immature cell markers in oral mucosa epithelial cells and rOMYCs**. K14 and p63 are immature cell markers of oral mucosal epithelium. Pax7 and CD34 are immature cells markers of rOMYCs. GAPDH was used as an internal control. GAPDH, glyceraldehyde-3-phosphate dehydrogenase; rOMYCs, rabbit oral myoblasts.

To identify the presence of highly proliferative cells in the epithelial and muscular layers of the three-layered laminated sheets, we performed RT-PCR on factors expressed in undifferentiated cells in each layers. In the three-layered sheets, temporal expression of K3 was observed in the oral mucosa epithelium, and proliferation-related K14 and p63, which are expressed in the epithelial basement membrane layer, were also observed consistently over time (Figure 5). We also observed sustained expression of desmin and of PAX7 and CD34, which are expressed in undifferentiated myoblasts (Figure 5).

## Discussion

In the present study, we fabricated three-layered sheets by culturing epithelial, mesenchymal, and myoblast cells isolated from rabbit oral mucosa for the purpose of developing cultured three-layered grafts containing a muscle layer. In particular, mesenchymal cells derived from connective tissue have a high proliferation rate upon subcultivation and are multipotent; furthermore, oral mucosa-derived mesenchymal stem cells have been reported to be multipotent in humans.^16, 17, 18^ We observed the expression of immature markers and desmin in isolated myoblasts (Figure 1). Moreover, previous studies have confirmed the existence of multipotent myoblasts similar to stem cells in skeletal muscle and surrounding areas.^19, 20^ Thus, it is possible that the myoblasts isolated in the present study may contain stem cells.

Laminating the five- to six-layer epithelium to the muscle sheet resulted in stratification to several times the original thickness, similar to what is observed *in vivo*. Myoblasts that undergo minimal stratification in regular cultures were also induced to stratify over time following lamination, and the expression of desmin also increased (Figure 3). There were no significant differences in the expression of either desmin or K13, which tended to be lower at day 7 compared with day 5, as shown in Figure 3. It is possible that stratification of the epithelial or myoblastic layers would affect the ability to observe reduced cytoskeletal filament protein expression through the entire hybrid sheet, as K4 and K13 are not expressed in the myoblastic layer, and desmin expression is limited to the myoblastic layer. In this study, we analysed temporal changes in the samples *ex vivo* for up to 7 days. Many previous studies have utilized longer sample incubation periods,^21, 22^ and constructs in these studies were very immature during the first weeks of development. Studies conducted over longer time periods are still required to examine the cellular maturation process. Furthermore, *in vivo* studies are needed to address the issue of potential carcinogenesis in an effort to demonstrate that hybrid sheets can be used for functional recovery of oral muscular defects. It is important that transplantable constructs be developed that permanently retain immature cells to provide necessary factors for tissue development.

Mesenchymal feeder cells are needed to produce a stratified epithelium, and are thought to play a role in maintaining undifferentiated cells.^23, 24, 25, 26^ Feeder cells can engage in direct cell-to-cell interactions or indirect interactions through growth factors or other factors, and studies have shown that improved epithelia stratification results from these interactions.^27, 28^ We did not observe epithelial or muscular stratification in two-layered epithelial–muscular sheets (data not shown), suggesting that the presence of an intervening mesenchymal cell-containing collagen layer, as in living tissues, may affect their stratification.

The thickness of both the epithelial and myoblastic layers in the constructs tended to plateau after day 5 (Figure 2). The size of viable constructs was limited by hypoxia, nutrient insufficiency, and waste accumulation due to poor vascularization. To overcome these limitations, Shimizu *et al.* fabricated functional, vascularized myocardial tissues using multistep transplantation by cell sheet integration *in vivo*.^29^ Although we did not investigate the effect of vascular growth on construct thickness, we believe that co-culture of vascular cells and prompt induction of vascularization may favorably affect construct thickness.

It is likely that immature cells with the potential to continue proliferating long after transplantation need to be present in each graft layer for successful engraftment and maintenance of homeostasis *in vivo*. Gene expression analysis of the three-layered sheets created in the present study indicated the presence of immature cells in both the epithelial layer and the muscle layer. Pax7 and CD34 are useful markers of skeletal muscle satellite cells (skeletal muscle stem cells), and co-expression of these markers on the surface of an immature myofibre is indicative of myogenesis from a satellite cell to a myofibre.^30, 31, 32^ It is possible that immature cells, such as satellite cells, were present in the constructs produced herein.

Garzon *et al.* reported that K8 and K19 are expressed in normal human oral mucosa and embryonic oral tissues.^33^ These proteins are key cytoskeletal constituents, and the monolayered oral mucosa constructs reported by Garzon *et al.* did not express these keratins.^33^ This suggests that their constructs exhibited keratin expression patterns similar to the non-keratinized human adult oral mucosa. We also did not observe the expression of K8, K19, and K10 in our construct by RT-PCR (data not shown).

The presence of K15-expressing immature phenotype cells has been reported in engineered epithelial sheets using an extra duplex feeder system, such as epithelial sheets from human corneal limbus.^28^ It has also been reported that mesenchymal bone marrow cells create a niche environment for maintaining hematopoietic stem cells.^34, 35^ It is thus conceivable that coculturing with mesenchymal cells also affects the maintenance of undifferentiated cells.

The expression of keratin and Coll IV, which was observed to increase with immunostaining, showed no temporal changes according to the western blotting analysis. This may be because the increased staining was concomitant with an increase in cell number, indicating that cellular concentrations were likely unaltered. Bustos *et al.*^36^ showed that cytokines that regulate inflammation, angiogenesis, epithelialization, matrix remodeling, and deposition were secreted from oral fibroblasts seeded onto collagen I scaffolds and these secretions were affected by cellular alignment and distribution, as well as the fiber orientation of the collagen microstructure. Although we have used collagen I gel as a substrate for oral mucosa epithelial cells and mesenchymal cell scaffolds in this study, most of the gel layer was positive for Coll IV, whereas the epithelial basement membrane was not strongly stained (Figure 4b). The microstructure of the collagen I gel might have affected Coll IV expression in the three-layer sheets. The expression of Coll IV in the epithelial basement membrane and muscle layers is important for immature epithelial cells and myoblasts as a niche for oral epithelial stem cells and satellite cells.^37, 38, 39, 40^ The expression of Coll IV among stratified myoblasts suggests that it has a role in myoblast proliferation and differentiation.

In the muscle layers of the three-layered sheets we created, we identified myotube-like structures that were also observed in high-density cultures of isolated myoblasts (Figure 4e). This suggests that extending the incubation period could result in the appearance of three-dimensional myotubes in the muscle layer of the three-layered sheets.

Future studies are needed to determine the direct and indirect effects of undifferentiated mesenchymal cells on the epithelial and muscle layers, and to assess the functional performance of the cultured grafts, including their capacity for exerting an epithelial defense function. Nevertheless, this is the first report of the successful creation of a three-layered epithelial–mesenchymal–muscular oral mucosa sheet that resembles oral mucosa *in vivo* and contains cells with the potential to proliferate after grafting. The three-layered sheets we developed here show promise as a new approach for re-establishing oral cavity shape and function when reconstructing the oral cavity.

## Figures and Tables

**Table 1:** Primer sequences and product size of RT-PCR

Protein	Primer	Sequence (5'→3')	Product size/bp
K3	Type II intermediate filaments in mammalian cells during differentiation and stratified epithelial cells	GACAATAATCGTTCCCTGGTTGCGGTAGGTGGCGATCT	434
K14	Type I intermediate filaments in inmature cells of stratified epithelial cells	ACTACCTGCAGCCGCCAGTTCAGTTCTTGGTGCGAAGGAC	1 417
p63	Tumour suppressor gene in inmature cells of stratified epithelial cells	CAGACTCAATTTAGTGAGGTGCCCCAACCATGAGCT	440
Pax7	Paired box transcription factor family member involved in maintaining proliferation and preventing differentiation in skeletal muscle progenitor cells	ATCCGGCCCTGTGTCATCTCCACGCGGCTAATCGAACTCA	278
CD34	Hematopoietic stem cell marker. CD34 expressed in quiescent adult skeletal muscle satellite cells	AATCTAGCCCAGTCTGAGGTCTTTCGGGAATAGCTCTGGT	174
Desmin	Type III intermediate filament in skeletal, smooth and cardiac muscle tissue	TGCAGGAGCTCAATGACCTCGATGCGAGCTAGTGTG	337
MyoD	A protein with a key role in regulating muscle differentiation	GCTCGCGAGGATGAGCATGTATGGCGTTGCGCAGGATCTC	239
GAPDH	Glyceraldehyde-3-phosphate dehydrogenase, internal control	ACCACAGTCCATGCCATCACTCCACCACCCTGTTGCTGTA	452

RT-PCR, reverse transcriptase polymerase chain reaction.
